# Integration between constrained optimization and deep networks: a survey

**DOI:** 10.3389/frai.2024.1414707

**Published:** 2024-06-19

**Authors:** Alice Bizzarri, Michele Fraccaroli, Evelina Lamma, Fabrizio Riguzzi

**Affiliations:** ^1^Department of Engineering, University of Ferrara, Ferrara, Italy; ^2^Department of Mathematics and Computer Science, University of Ferrara, Ferrara, Italy

**Keywords:** deep learning, symbolic artificial intelligence, constrained training, constrained neural architecture search, neural-symbolic integration

## Abstract

Integration between constrained optimization and deep networks has garnered significant interest from both research and industrial laboratories. Optimization techniques can be employed to optimize the choice of network structure based not only on loss and accuracy but also on physical constraints. Additionally, constraints can be imposed during training to enhance the performance of networks in specific contexts. This study surveys the literature on the integration of constrained optimization with deep networks. Specifically, we examine the integration of hyper-parameter tuning with physical constraints, such as the number of FLOPS (FLoating point Operations Per Second), a measure of computational capacity, latency, and other factors. This study also considers the use of context-specific knowledge constraints to improve network performance. We discuss the integration of constraints in neural architecture search (NAS), considering the problem as both a multi-objective optimization (MOO) challenge and through the imposition of penalties in the loss function. Furthermore, we explore various approaches that integrate logic with deep neural networks (DNNs). In particular, we examine logic-neural integration through constrained optimization applied during the training of NNs and the use of semantic loss, which employs the probabilistic output of the networks to enforce constraints on the output.

## 1 Introduction

Artificial intelligence (AI) has long been characterized by two primary paradigms: symbolic and neural approaches. The field of AI has witnessed notable advancements since its inception in the 1950s. Seminal works by McCulloch and Pitts (McCulloch and Pitts, 1943) laid the groundwork for neural networks, while Turing's seminal contributions in the 1950s introduced the concept of machine intelligence (Turing, 2009). Symbolic AI dominated the landscape until the 1980s, after which neural AI began to grow and attract considerable attention. The ongoing debate about these two approaches remains extensive. However, in recent years, the convergence of symbolic and neural AI methodologies has gained traction. In addition to fundamental symbolic or neural techniques, hybrid applications incorporating both symbolic and neural features have emerged.

The main differences between the neural and symbolic fields of AI are as follows:

1. Neural approaches provide associative results, while symbolic approaches produce logical conclusions
2. Neural methods learn and adapt to the data provided, whereas human intervention is common in symbolic methods
3. Neural methods, such as deep learning (DL) that use multiple layers to progressively extract higher-level features from the raw input Deng et al. (2014), can handle large and noisy datasets, while symbolic methods perform better when dealing with relatively small and precise data.

The popularity of machine learning (ML) in recent years has raised questions about which tasks are suitable for deep learning, which ones require model-based symbolic reasoning, and what advantages an integration between the two approaches might bring.

Several algorithms that bridge symbolic and neural methodologies have been developed, including knowledge-based neural networks (KBNN or KBANN) (Agre and Koprinska, 1996), graph neural networks (GNNs) (Lamb et al., 2020), connectionist logic programming and inductive learning (C-IL^2^P) (Avila Garcez and Zaverucha, 1999), connectionist knowledge time logic (CTLK) (Garcez et al., 2019), the hybrid expert system (HES) (Sahin et al., 2012), and the tensor product representation (Smolensky, 1990), which features a neural network core coupled with a symbolic problem solver.

This study aims to analyze the integration of constraints in a neural context from two perspectives, which, although seemingly different, share common features: constrained neural architecture search (NAS) and the integration of constraints in neural networks. Both paradigms focus on incorporating restrictions and prior knowledge into the process of building and training neural networks to improve efficiency, interpretability, and compliance with specific application requirements. Both require the incorporation of knowledge and the use of constrained optimization, although for different purposes. In constrained NAS, constraints are applied during the architecture search phase to create neural networks optimized for specific contexts, generally within the realm of TinyML, which involves physical constraints such as computational limitations. In the second case, constraints are applied during the training of the network itself to achieve networks that perform better in specific contexts, such as handling imbalanced data, or to enhance network performance by injecting domain knowledge.

In this study, after providing necessary background information, we delve into the state-of-the-art integration of NAS with constraints dictated by the physical limitations inherent in embedded systems, a domain commonly referred to as Tiny Machine Learning (TinyML). NAS methodologies have gained increasing popularity (Benmeziane et al., 2021) and have become indispensable for expediting and automating the arduous and error-prone process of synthesizing novel DL architectures. While NAS has been extensively researched in recent years and has exhibited remarkable success, its practical applicability to real-world challenges still poses significant hurdles. Notably, the complexity of convolutional neural network architectures makes them unsuitable for deployment on resource-constrained platforms typical of TinyML, such as mobile and embedded systems.

Within this survey, we illustrate various solutions from the literature aimed at adapting NAS systems for TinyML. Specifically, we examine several NAS frameworks (Dong et al., 2018; Zhou et al., 2018; Jin et al., 2019; Tan et al., 2019; Fraccaroli et al., 2021, 2022; Liberis et al., 2021) and explore potential methodologies for incorporating physical constraints into synthesized networks.

In the second part of the survey, we scrutinize works where contextually inferred constraints are leveraged to enhance neural network performance. We present examples of constrained neural networks (NNs) employing penalty methods, such as the work by Sangalli et al. (2021), where a deep neural network (DNN) is formulated for binary classification under class imbalance conditions as a constrained optimization problem, alongside novel frameworks for out-of-distribution (OOD) detection (Katz-Samuels et al., 2022). Then, we exemplify probabilistic integration with the study conducted by Xu et al. (2018), where a novel methodology is proposed for integrating symbolic knowledge into deep learning. They derive a semantic loss function that establishes a connection between neural output vectors and logical constraints, taking into account the extent to which the neural network adheres to the constraints imposed on its output.

This study is structured as follows: Section 2 offers an overview of fundamental concepts encompassing deep neural networks (DNN), automated machine learning (AutoML), optimization, and constraints. Section 3 delves into different approaches to NAS, multi-objective optimization (MMO), and their integration. Section 4 explores the integration of logic and deep learning through the Lagrange Multiplier Method and probabilistic interpretation, along with their practical applications. Finally, in Section 5, we draw conclusions based on the findings presented.

## 2 Main concepts

This section provides an overview of the basic components of the most popular DNNs. Then, it presents the main research concepts of autoML, optimization, and constraints, to provide a general overview of the topics covered in the following sections.

### 2.1 DNNs

Recently, DNNs are one of the hottest areas of ML. Their application spans across diverse domains, including Computer Vision (CV), Natural Language Processing (NLP), and robotics. DNNs, or neural networks with multiple hidden layers, possess the capability to automatically extract features from extensive unstructured datasets, such as text, images, and audio, or from large tabular data (e.g., clinical data). Through their multi-layered architecture, DNNs can iteratively learn the mapping between input features and predicted classes, achieving high levels of accuracy.

Given their versatility, DNNs find extensive applications in various domains. Notably, in CV, convolutional neural networks (CNNs) emerge as the primary tool, leveraging convolutions to extract crucial feature vectors from input images. Prominent examples include AlexNet (Krizhevsky et al., 2017), VGG (Simonyan and Zisserman, 2014), GoogLeNet (Szegedy et al., 2015), ResNet (He et al., 2016), and DenseNet (Huang et al., 2017). In the realm of CV, networks are typically classified into three main categories based on the task they perform: image classification (He et al., 2016; Howard et al., 2017; Tan and Le, 2019; Dai et al., 2021), object detection (Redmon et al., 2016; Lin et al., 2017), and semantic segmentation (Badrinarayanan et al., 2017; He et al., 2017).

Another significant application domain is NLP, renowned for its complexity owing to the inherent ambiguity characteristic of human language. In this study, recurrent neural networks (RNNs) such as Long Short-Term Memory (LSTM) (Staudemeyer and Morris, 2019) and Gated Recurrent Unit (GRU) (Cho et al., 2014), alongside NNs with memory, are employed to learn context and word connections. In recent years, the rise of transformers (Tenney et al., 2019; Lin et al., 2021) has revolutionized NLP, with their self-attention mechanism enabling the capture of relationships among words in a sentence. Notably, transformers have also found application in CV, demonstrating state-of-the-art performance (Dai et al., 2021).

### 2.2 AutoML

There are many different ML algorithms, each characterized by a distinct set of hyperparameters, resulting in an overwhelming array of potential alternatives. Consequently, their application typically entails a complex endeavor necessitating experience, time, and labor. AutoML (He et al., 2021), an emerging scientific discipline, addresses this challenge by exploring methodologies for the efficient, objective, and data-driven construction of ML models.

In recent years, many approaches have been proposed for both the construction and optimization of model learning pipelines and the development of DNNs. AutoML methods can be categorized on the basis of various criteria, including the optimization method employed (e.g., Bayesian optimization, genetic programming, and random search), the structure of the generated pipelines (e.g., with or without fixed structure), and the utilization of meta-learning for leveraging insights from prior datasets or post-processing tasks such as ensemble construction (Gijsbers et al., 2019). Noteworthy examples of AutoML frameworks include Auto-WEKA (Thornton et al., 2013), auto-sklearn (Feurer et al., 2015), and AutoKeras (Jin et al., 2019).

The following section provides a succinct overview of the foundational principles underlying all AutoML systems.

#### 2.2.1 Automated HPO

Automated hyperparameter optimization (HPO) is a crucial task within AutoML systems, particularly for optimizing the hyperparameters (HPs) of ML algorithms, including DNNs, that are particularly sensitive to the choice of hyperparameters, making automated HPO essential for achieving optimal performance. The significance of automated HPO is underscored by its multifaceted utility:

- **Reduction of human effort:** Automated HPO alleviates the burden on human practitioners by automating the tedious and time-consuming process of manually tuning hyperparameters for ML applications.
- **Performance enhancement:** Through automated optimization, ML algorithms can achieve improved performance, leading to new state-of-the-art results across various machine learning tasks (Snoek et al., 2012; Melis et al., 2017).
- **Enhanced reproducibility and fairness:** Automated HPO facilitates fair comparisons between different ML methods by ensuring that they are all evaluated under the same hyperparameter configurations. This enhances the reproducibility and fairness of scientific studies (Bergstra et al., 2013).

However, HPO presents several challenges that make it a difficult problem. The main challenges includes: the cost of evaluating functions for large models, complex machine learning pipelines, or large datasets; the complexity and high density of the configuration space (encompassing a mix of continuous, categorical, and conditional hyperparameters); the lack of access to the gradient of the loss function with respect to HPs; and the inability to directly optimize generalization performance due to the limited size of the training datasets (Feurer and Hutter, 2019).

The Automated HPO problem can be formally defined as follows:

Let A denote a machine learning algorithm with *N* HPs. We denote the domain of the *n*-th HP by Λ_*n*_ and the overall HP configuration space as **Λ** = Λ_1_ × Λ_2_ × ⋯ × Λ_*n*_. A vector of hyperparameters is denoted by λ ∈ **Λ**, and A with its hyperparameters instantiated to λ is denoted by $A_{\lambda }$. Given a dataset D, our goal is to find:


(1) $$\begin{array}{l} \lambda^{*}=\underset{\lambda \in \Lambda }{arg\#x000A0;min}\;{𝔼}_{\left(D_{train},D_{valid}\right)\sim D}\text{V}\left(L,A_{\lambda },D_{train},D_{valid}\right), \end{array}$$

where $\text{V}\left(L,A_{\lambda },D_{train},D_{valid}\right)$ measures the loss of a model generated by algorithm A with hyperparameters λ on training data *D*_*train*_ and evaluated on validation data *D*_*valid*_. In real applications, we have access to a limited number of D~D data; therefore, it is necessary to approximate the expectation in Equation (1).

Popular choices for the validation protocol **V**(·, ·, ·, ·) are holdout error and cross-validation for a user-given loss function (such as the misclassification rate). Several strategies have been proposed to reduce evaluation time: it is possible to test ML algorithms only on a subset of the folds (Thornton et al., 2013), a subset of data (Swersky et al., 2013; Klein et al., 2017), or for a limited number of iterations.

#### 2.2.2 Meta-learning

Meta-learning, or learning to learn, is the science of systematically studying how different ML systems perform on a wide range of tasks to learn from this experience (*meta-data*) and perform a new task as quickly as possible. This not only allows the user to improve the performance of the ML design but also replaces hand-tuned algorithms with new algorithms learned in a data-driven way (Vanschoren, 2019). First, it is necessary to record the exact algorithm configurations used for training the models (e.g. HPs, pipelines, network architectures, and training time), that is, the *meta-data* describing previous training activities. Then, we have to learn from these *meta-data* to extract and transfer knowledge to new tasks. The term *meta-learning* refers to any type of learning based on previous experiences with other tasks. The more similar the previous tasks are, the more types of *meta-data* we can exploit. When a new task represents completely unrelated phenomena, exploiting previous experiences will not be effective.

#### 2.2.3 NAS framework

In recent years, NAS frameworks have become fundamental for optimizing neural network HP. NAS, a subfield of AutoML, exhibits significant overlap with HPO and meta-learning methodologies.

NAS aims at discovering the best neural network architecture for specific requirements. It encompasses a suite of tools and methodologies that systematically explore and evaluate many architectures within a predefined search space, employing various search strategies such as random search, grid search, genetic algorithms, or Bayesian search (Elsken et al., 2019; Liashchynskyi and Liashchynskyi, 2019) to select the most promising candidate.

A NAS framework can be divided into three components. The first component is the search space that defines which architectures are allowed; it can be reduced by incorporating prior knowledge about the properties that architectures must have to be suitable for a task. Second, the search strategy indicates how the NAS algorithm explores the search space to find optimal or near-optimal architectures. Finally, the performance estimation strategy refers to the process of estimating the performance of the generated networks: the simplest option is to perform training and validation of the architecture on the data.

NAS generally aims to achieve the best test accuracy without considering the computational cost of inference thus, the generated networks might not be suitable for embedded systems.

In Talbi (2021), the authors propose a unified method for describing various optimization algorithms that focus on the common and important search components of optimization algorithms. They also extend this unified methodology to advanced optimization approaches, such as surrogate, multi-objective, and parallel optimization. The authors propose several constraint management strategies distinguishing them into categories as follows:

1. Rejection: only feasible solutions are retained during the optimization process, and infeasible solutions are automatically discarded (Dong et al., 2018; Hsu et al., 2018; Liberis et al., 2021).
2. Penalization: all solutions are considered, but those that are not feasible are penalized. The objective function is extended by a penalty function. This is the most popular approach, in which many alternatives have been used to define penalties (Veniat and Denoyer, 2018; Zhou et al., 2018; Tan et al., 2019; Liberis et al., 2021).
3. Repair: heuristic algorithms that transform an unfeasible solution into a feasible solution (He and Sun, 2015; Tan and Le, 2019).
4. Preserving: strategies that incorporate problem-specific knowledge into encoding and search operators to generate only feasible solutions. This can reduce the size of the search space and thus simplify the search process (Lu et al., 2018; Wang et al., 2018).

### 2.3 Optimization

Optimization is a branch of applied mathematics that studies the theory and methods for finding the maximum and minimum points of a mathematical function within a specified domain.

A simple example of an optimization problem is maximizing or minimizing a real function of a real variable over a given interval. More generally, optimization involves finding a sufficiently optimal value for some objective function in a given domain (or input). Adding more than one objective to an optimization problem increases its complexity. In this case, we have a multi-objective optimization (MOO).

#### 2.3.1 MOO

A MOO problem is defined as follows (Equation 2):


(2) $$\begin{array}{l} \underset{x\in X}{\mathrm{minimize}}F\left(x\right)={\left[f_{1}\left(x\right),f_{2}\left(x\right),\ldots ,f_{n}\left(x\right)\right]}^{T} \end{array}$$

where the integer *n* ≥ 2 is the number of objectives, and *X* ⊆ ℝ^*n*^ is the feasible set (Hwang and Masud, 2012).

Due to their conflicting nature, all objectives cannot be optimized simultaneously. Consequently, most MOO approaches aim at recovering the Pareto front, which can be defined as the set of Pareto optimal points. A point is considered Pareto optimal if it cannot be improved in any of the objectives without degrading another objective.

MOO methods can be essentially divided into two classes:

1. Methods that generate single points that are candidates to be Pareto points;
2. Methods that generate optimal approximation of the Pareto front,

In the first class, a reference vector *z*^*ref*^ ∈ ℝ^*n*^ in the space of objectives is defined, and a solution in the space of variables is determined to minimize the distance between the vector of objective functions and the reference (Miettinen, 1999). Typically, one uses the ideal vector of goals Z^*^ defined by its components as a reference vector (Equation 3):


(3) $$\begin{array}{l} Z_{i}^{*}=\underset{x\in X}{\min}f_{i}\left(x\right) \end{array}$$

Therefore, we can define the function that minimizes the distance between the value of the multi-objective function *F*(*x*) and the ideal vector Z^*^ as the goal function (Equation 4):


(4) $$\begin{array}{l} \underset{x\in X}{\min}||F\left(x\right)-Z^{*}|| \end{array}$$

In the second class, a representative approach is known as the method of weights. Consider the following problem (Equation 5):


(5) $$\begin{array}{l} \underset{x\in X}{\min}\overset{N}{\underset{i=1}{\sum }}w_{i}f_{i}\left(x\right) \end{array}$$

where the weights *w*_*i*_ are such that *w*_*i*_ > 0 and $\overset{N}{\underset{i=1}{\sum }}w_{i}=1.$ As the vector of weights *w* varies, different Pareto points can be obtained.

### 2.4 Integration of constraints and DL

One of the main challenges of ML models is the satisfaction of physical constraints. Without these constraints, ML models, while optimizing the loss function, may deviate from known physical principles. Constraints can be applied in two ways: soft constraints and hard constraints. The latter must be satisfied by any feasible solution model. On the other hand, a soft constraint can be violated, but violation of the constraint results in a penalty in the objective function (often, the greater the amount by which the constraint is violated, the greater the penalty). ML models with hard constraints have some advantages over soft constraints, such as more robust and accurate predictions, but are usually difficult to optimize due to the strict adherence to constraints.

Integrating constraints with DL presents a challenge because logic is discrete, symbolic, and semantic, while DL is continuous, smooth, and differentiable. However, there exist different methods for the integration, such as penalty, Lagrange multipliers, and probabilistic interpretation.

#### 2.4.1 Penalty

Penalty methods are algorithms for finding local minima or maxima of a function subject to constraints. They transform the problem with constraints into a problem or problems of unconstrained optimization. The unconstrained problems are formed by adding a term, the *penalty function*, to the objective function that consists of a *penalty factor* multiplied by a measure of violation of the constraints. The measure of violation is non-zero when the constraints are violated and becomes zero in the region where the constraints are not violated.

Consider the following optimization problem (Equation 6):


(6) $$\begin{array}{l} \begin{array}{c} \mathrm{minimize}f\left(\theta \right)\\ \text{subject to}:c\left(\theta \right)\leq 0 \end{array} \end{array}$$

where *f*:ℝ^*n*^ → ℝ is the objective function, *c*:ℝ^*n*^ → ℝ^*c*^ is the constraints function. Penalty methods replace the problem with one or more problems without constraint of form (Equation 7):


(7) $$\begin{array}{l} \mathrm{minimize}\Phi \left(\text{x}\right)=f\left(\text{x}\right)+\phi g\left(c\left(\theta \right)\right) \end{array}$$

where ϕ is a *penalty factor* and *g*(·) is the *penalty function*.

#### 2.4.2 Lagrange multipliers

Let θ_0_ be an optimal solution to the following optimization problem (Equation 8):


(8) $$\begin{array}{l} \begin{array}{c} \mathrm{minimize}f\left(\theta \right)\\ subject\;to:c\left(\theta \right)=0 \end{array} \end{array}$$

where *f*:ℝ^*n*^ → ℝ is the objective function, and *c*:ℝ^*n*^ → ℝ^*c*^ is the constraints function, both having continuous first derivatives. We introduce a new variable (λ) called a Lagrange multiplier and study the Lagrange function defined by Equation (9):


(9) $$\begin{array}{l} L\left(\theta ,\lambda \right)=f\left(\theta \right)-\lambda c\left(\theta \right) \end{array}$$

The Lagrange multipliers theorem states that if *f*(θ_0_) is a minimum of *f*(θ) for the original constrained problem and ∇*c*(θ_0_) ≠ 0, then there exists a λ_0_ such that $\nabla L\left(\theta_{0},\lambda_{0}\right)=0$, i.e., (θ_0_, λ_0_) is a stationary point for the Lagrange function. Lagrange multiplier methods generate a class of algorithms for solving constrained optimization problems [e.g., the Augmented Lagrangian method (Bertsekas, 1997)].

#### 2.4.3 Probabilistic interpretation

Another way to integrate constraints with DL is to use a probabilistic interpretation. The output of a NN is a probability distribution over the classes (in the case of classification). If we have constraints on the output, we can measure how close the output is to satisfying these constraints. Let the NN output be (*x*_0_, *x*_1_, *x*_2_) ∈ [0, 1] and the constraint be that exactly one of these values should be true (i.e., one-hot encoding):


(10) $$\begin{array}{l} 𝔼xactly-one=\{\begin{array}{c} a\wedge \neg b\wedge \neg c\\ \vee \\ \neg a\wedge b\wedge \neg c\\ \vee \\ \neg a\wedge \neg b\wedge c \end{array} \end{array}$$

where *a, b, c* ∈ {0, 1}. Then, the probability that the constraint in Equation (10) is satisfied is given by Equation (11):


(11) $$\begin{array}{l} \begin{array}{c} x_{0}\left(1-x_{1}\right)\left(1-x_{2}\right)+\\ \left(1-x_{0}\right)x_{1}\left(1-x_{2}\right)+\\ \left(1-x_{0}\right)\left(1-x_{1}\right)x_{2} \end{array} \end{array}$$

In other words, we can measure the probability that the constraint is satisfied given the output of the network.

## 3 Constrained neural architecture search

The purpose of this section is to illustrate different NAS approaches and their possible integration to generate NNs that meet the physical constraints required by embedded systems (e.g., latency, memory, and energy). Therefore, we mainly see penalization and rejection strategies. We also consider AutoML as a multi-objective problem in which many different and conflicting objectives are optimized.

### 3.1 Approches to NAS

In Elsken et al. (2019), Elsken et al. provide an overview of existing work in this field, classifying it along three dimensions: search space, search strategy, and performance estimation strategy.

Morphism-based NAS systems start from a basic network and, iteration after iteration, modify its structure, including changes in depth, width, kernel size, and even subnetworks. In this survey, we will focus on three types of morphism-based NAS systems, looking at an example for each:

a) An AutoML system that makes several search strategies available (Jin et al., 2019).
b) A symbolic tuner that exploits symbolic rules and Bayesian optimization to explore the search space (Fraccaroli et al., 2021, 2022);
c) Some tuners for microcontroller systems that uses multi-objective optimization (Dong et al., 2018; Tan et al., 2019; Liberis et al., 2021).

AutoKeras (Jin et al., 2019), an open source system based on Keras, is an example of the AutoML system that offers several search strategies. The goal is to allow domain experts who are not familiar with machine learning technologies to easily use machine learning techniques. AutoKeras provides several tools to define and explore the search space through different algorithms (e.g., Bayesian optimization, random search, and grid search) and strategies (e.g., rejection and penalization). Symbolic DNN-Tuner (Fraccaroli et al., 2021) uses Bayesian optimization (BO) that is the state-of-the-art HPO algorithm for DL. BO keeps track of past results and uses them to build a probabilistic model, constructing a probability density of the HP space. Symbolic DNN-Tuner (Fraccaroli et al., 2021) aims to improve BO applied to DNNs through an analysis of network results on training and validation sets. The system applies symbolic tuning rules, implemented in probabilistic logic programming (PLP) (Riguzzi, 2022). The results obtained from the training and validation phases are logically evaluated, and by applying symbolic tuning rules, the network architecture and its HPs are corrected, leading to improved performance.

**Figure 1:**
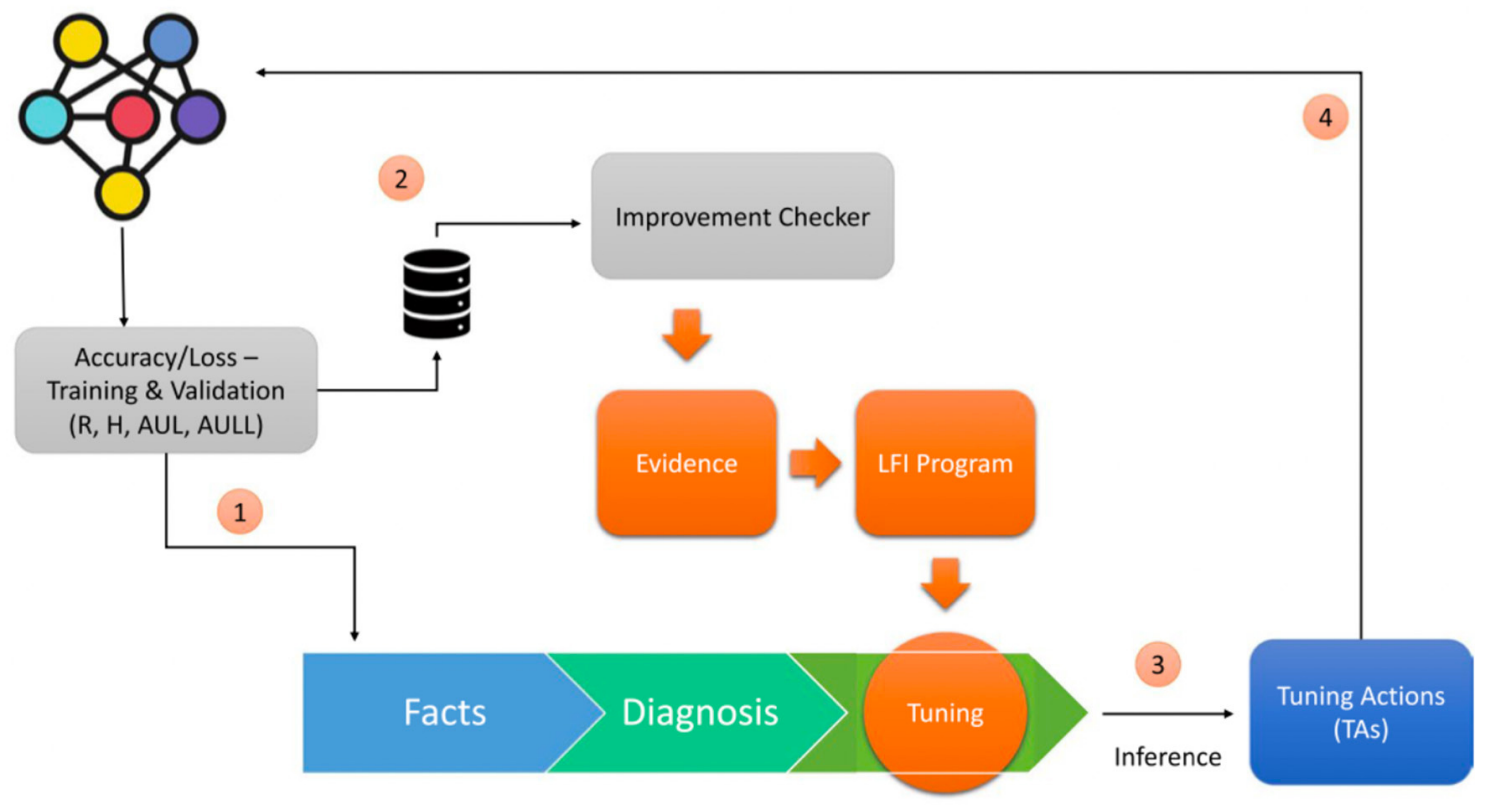
Symbolic DNN-tuner architecture [reprinted with permission from Fraccaroli et al. (2021)].

Figure 1 shows the architecture of Symbolic DNN-Tuner where the neural block returns values from the trained network that are passed both to the Improvement Checker (2), which checks the improvement of the network, and to the symbolic program (1) consisting of three parts: facts, which store the data obtained from the neural block; diagnosis, which analyzes the behavioral problems of DNNs; and tuning, which is composed of the Symbolic Tuning Rules. Using ProbLog (De Raedt et al., 2007) inference, it is possible to query this program and obtain the tuning actions (TAs) (3). The TAs are then passed to the neural block and applied to the DNN structure or HP search space (4).

Finally, μNAS (Liberis et al., 2021) is an example of a NAS for microcontrollers. This system focuses on using deep learning to add computational intelligence to small personal IoT devices. This would allow computations to be performed locally, ensuring that the user's data remains on the device. As a result, it achieves a greater degree of privacy and autonomy. IoT devices are powered by microcontroller units (MCUs). MCUs are ultra-small computers with very limited resources contained in a single chip. This allows MCUs to be cheaper and more energy-efficient than desktop devices or cell phones. However, these advantages lead to drastically reduced computing power. In Liberis et al. (2021), the authors propose the use of a reduced search space that adheres to the constraints on the limited resources and a multi-objective function to explore the search spaces. The MOO aims to find the optimal parameters α^*^ defined as follows (Equation 12):


(12) $$\begin{array}{l} \alpha^{*}=\underset{\alpha \in S}{\arg\min}ℒ(\alpha )\\ \;=\underset{\alpha \in S}{\arg\min}(1.0-ValAccuracy(\alpha ),\\ \;ModelSize(\alpha ),\\ \;PeakMemUsage(\alpha ),\\ \;Latency(\alpha )) \end{array}$$

where *ValAccuracy* is a measure of the quality of the network, *ModelSize* is the size of the network, *PeakMemUsage* is the maximum number of parameters stored at a time, and *Latency* is the time it takes to perform a single inference. The Liberis and Lane (2019) algorithm was used to compute the maximum number of stored parameters. To optimize the multiobjective function, the authors used the method introduced by Paria et al. (2020).

In Liberis et al. (2021), the authors showed that, with proper design of the search space and explicit identification of physical constraints, it is possible to create a NAS system that discovers resource-efficient models for a variety of image classification tasks. Table 1 shows the main results of the μNAS compared with other constrained NAS. They use multiply accumulate operations (MACs) as a measure to quantify the latency time as a function of the model size.

**Table 1:** Pareto-optimal architectures discovered by μ*NAS* vs. other Resource—Constrained NAS.

**Dataset**	**Model**	**Acc. (*%*)**	**Model size**	**MACs**
MNIST	SpArSe (Fedorov et al., 2019)	98.64	2770	-
	BonsaiOpt (Kumar et al., 2017)	94.38	490	-
	ProtoNN (Gupta et al., 2017)	95.88	63′900	-
	μ*NAS*(1174 steps, 1 GPU-day)	99.19	480	28.6 K
CIFAR-10	LEMONADE (Elsken et al., 2018)	≈91.77	10K	-
	μ*NAS*(4205 steps, 23 GPU-days)	86.49	11.4 K	384 K
Speech	RENA (Zhou et al., 2018)	94.04	47 K	≈700*M*
Commands	DS-CNN (Zhang et al., 2017)	94.45	<38.6 K	≈2.7*M*
	MCUNet (Lin et al., 2020)	91.20	<1 M	-
	μ*NAS*(1960 steps, 39 GPU-days)	95.36	37 K	1.1 M

Other examples of physically constrained NAS using MOO can be found in the literature. For example, in Dong et al. (2018), the authors propose Device-aware Progressive Search for Pareto-optimal Neural Architectures (DPP-Net), which optimizes device-related and device-independent targets by applying multi-objective optimization. DPP-Net employs a compact search space inspired by state-of-the-art mobile CNNs and further improves the search efficiency by adopting progressive search (Liu et al., 2017). The authors test their system on CIFAR-10 and they compere it with the state-of-the-art (Huang et al., 2017, 2018; Zoph et al., 2018). We show these results in Table 2.

**Table 2:** DPP-Net main result on CIFAR-10.

**Model**	**Type**	**Error rate**	**Params**	**FLOPs**
DenseNet-BC (*k* = 12) (Huang et al., 2017)	Manual	4.51	0.8M	-
CondenseNet-86 (Huang et al., 2018)	Manual	5.0	0.52M	65.8M
NASNet-B (Zoph et al., 2018)	Auto	3.73	2.6M	-
DPP-Net (Dong et al., 2018)	Auto	4.36 ~ 5.84	11.39M ~ 0.45M	1364M ~ 59.27M

In Tan et al. (2019), the authors proposed an automated mobile neural architecture search (MNAS) approach that explicitly incorporates model latency into the main objective so that the search can identify a model that achieves an optimal trade-off between accuracy and latency. The authors define the objective function as follows:

Given a model *m*, let *ACC*(*m*) denote its accuracy on the target task, *LAT*(*m*) the inference latency on the target mobile platform, and *T* the target latency. A common method is to treat *T* as a hard constraint and maximize the accuracy under this constraint (Equation 13):


(13) $$\begin{array}{l} \begin{array}{c} \underset{m}{\mathrm{maximize}}\;ACC\left(m\right)\\ \text{subject to }\mathrm{LAT(m)}\leq T \end{array} \end{array}$$

Tan et al. (2019) use a custom weighted product method to approximate Pareto optimal solutions, with an optimization objective defined as Equation (14):


(14) $$\begin{array}{l} \underset{m}{\mathrm{maximize}}\;ACC\left(m\right)\times {\left[\frac{LAT\left(m\right)}{T}\right]}^{w} \end{array}$$

where *w* is an application-specific constant. The authors test their system on ImageNet classification and compare their model with both manually designed mobile models and other automated approaches. The results are shown in Table 3.

**Table 3:** MNAS main result on ImageNet classification.

**Model**	**Type**	**Top-1 Acc. (%)**	**Params**	**MACs**
MobileNetV1 (Howard et al., 2017)	Manual	70.6	4.2M	575M
SqueezeNext (Gholami et al., 2018)	Manual	67.5	3.2M	708M
ShuffleNet (1.5x) (Zhang et al., 2018)	Manual	71.5	3.4M	292M
ShuffleNetV2 (1.5x) (Ma et al., 2018)	Manual	72.6	5.4M	524M
CondenseNet (G=C=4) (Huang et al., 2018)	Manual	71.0	-	299M
MobileNetV2 (Sandler et al., 2018)	Manual	72.0	-	597M
NASNet-A (Zoph et al., 2018)	Auto	74.0	2.9M	274M
AmoebaNet-A (Zhou et al., 2018)	Auto	74.5	4.8M	529M
PNASNet (Liu C. et al., 2018)	Auto	74.2	3.4M	300M
DARTS (Liu H. et al., 2018)	Auto	73.1	6.9M	585M
MNAS	Auto	75.2 ~ 76.7	3.9M ~ 5.2	312M ~ 403M

Zhou et al. (2018) developed the Resource-Efficient Neural Architect (RENA), a resource-limited efficient NAS that uses reinforcement learning with network embeddings. The framework consists of a network of policies to generate actions that define the architecture of the neural network. The environment provides the performance of the trained neural network and its resource utilization. RENA uses a policy gradient with accumulated rewards to train the policy network. To find neural architectures that satisfy multiple resource constraints, a reward based on the model performance should be penalized according to the amount of constraint violation. A hard penalty may be effective for some constraints, but it would be difficult for the controller to learn from very sparse rewards under tight resource constraints. Therefore, RENA uses a soft continuous penalty method to find architectures with high performance while meeting all resource constraints. The results obtained with this framework are shown in Table 4.

**Table 4:** RENA main result on speech commands.

**Model**	**Resource constraint**	**Parameters**	**Accuracy (%)**	**FLOPs**
GRU (Zhang et al., 2017)	-	0.093 M	92.94	0.68 B
DS-CNN (Zhang et al., 2017)	-	0.023 M	93.39	6.07 B
CRNN (Zhang et al., 2017)	-	2.447 M	94.40	46.21 B
RENA	-	0.143 M	95.81	3.39 B
RENA	Model size < 0.05 M	0.047 M	94.04	1.40 B
RENA	Model size < 0.1 M	0.067 M	94.82	6.53 B
RENA	Comp. complexity < 1 GFLOPs	0.425 M	93.16	0.89 B
RENA	Comp. complexity < 5 GFLOPs Model size < 0.1 M	0.171 M	95.02	3.30 B
RENA	Comp. complexity < 1 GFLOPs	0.035 M	93.07	1.0 B

A popular case study is the application of machine learning models for the predictive maintenance of IoT edge devices in a factory. Since these devices have limited memory and computing resources, we need to design optimized neural architectures for them, striking a balance between model accuracy and resource efficiency. Using RENA, we are able to develop models that can effectively predict device failures while operating within the constraints of edge devices. The implementation of RENA therefore enables the company to use machine learning for the predictive maintenance of its IoT edge devices efficiently.

Another case study example are TinyML models designed specifically for wearable devices with limited memory and processing capacity. They are optimized using techniques such as pruning and quantization to save energy and resources while still providing accurate predictions. For example, on environmental monitoring sensors, a system such as μNAS can be applied to design compact neural architectures for low-power microcontrollers, enabling real-time data analysis and maximizing battery life. These examples highlight the effectiveness of constrained NAS approaches, such as RENA, DNN-Tuner, and μNAS, in designing neural architectures tailored to specific constraints.

### 3.2 Integrated system

The methods outlined above shed light on the distinctions between various NAS approaches. However, the question arises: can MOO methods be seamlessly integrated with each other? Moreover, is it feasible to introduce constraints without resorting to MOO?

One prospective avenue is to introduce into the Symbolic DNN-Tuner (Fraccaroli et al., 2021) a multi-objective function, akin to the μNAS model, which stands as a subject for future exploration. In such a scenario, the optimization function would be multi-objective in nature, aiming to optimize the network while considering physical constraints. This function could be optimized using one of the methods delineated in Section 2.3.1. Following this optimization, incorporating tuning ations (TAs) would enable the system to intervene when the networks violate the constraints. Table 5 illustrates the tuning actions employed by Symbolic DNN-Tuner (Fraccaroli et al., 2021), alongside potential additional rules to satisfy new constraints, presented in the lower part of the table.

**Table 5:** Problem, symptoms, and tuning actions.

**Problem**	**Symptoms**	**Tuning actions (TAs)**
Overfitting	Gap between accuracy or loss in training and validation	Regularization and Batch
		Normalization
		Increase dropout
		Data augmentation
Underfitting	High loss	Decrease the learning rate
	Low accuracy	Increase the number of neurons
		Addition of fully connected layers
		Addition of convolutional blocks
Increasing loss	Loss trend analysis	Decrease the learning rate
Fluctuating loss	Fluctuation of the loss	Increase the batch size
		Decrease the learning rate
Low learning rate	Evaluation of the shape of the loss	Increase learning rate
High learning rate	Evaluation of the shape of the loss	Decrease learning rate
Peak Memory Usage	High number of Parameters for layer	Decrease the number of neurons
Model Size	High number of total Parameters	Decrease the number of layers or the number of neurons
Latency	High FLOPS	Decrease the number of layers or the number of neurons

Another way to impose a constraint might be to add a penalty to the metric used to evaluate the networks. For example, AutoKeras adds a penalty term to the network loss. This means that the more a constraint is violated, the greater the penalty will be. In the previous example of physical constraints, assuming an upper limit on FLOPS, the closer the network to the limit, the greater the penalty. Thus, a NAS will tend to prefer smaller networks since large networks would lead to higher loss functions.

## 4 Constrained networks

The widespread success of DL has led several researchers to look for ways to improve it through constraint-based domain knowledge. There are contexts in which purely data-driven models are not ideal, such as when data are sparse or learning tasks are very challenging. It is possible to achieve a significant increase in the performance of NNs by exploiting domain knowledge, using problem-specific information for simplifying the training process (e.g., the shape of the output, the data generation process, the experience of a domain expert, etc.). Therefore, it makes sense to exploit domain information in order to not to start from scratch when tackling difficult learning tasks for NNs.

In various fields, constraints are applied during the training of neural networks to improve the performance and reliability of the models. In the case of unbalanced datasets, penalties for misclassification errors on minority classes are applied to address the issue (Sangalli et al., 2021). Anomaly detection systems could use constraints to penalize misclassification of normal cases as anomalies during the training process. Finally, medical diagnostic systems could incorporate medical constraints during training to ensure that the provided diagnoses align with clinical evidence and medical guidelines. These constraints play a crucial role in enhancing the effectiveness and validity of the neural network models in these respective domains.

Below, we will illustrate some applications of constrained NNs; in particular we will see examples of constraints applied through penalty functions, such as Lagrange multipliers (Section 4.1), applied in unbalanced data and OOD detection contexts. Finally, we will see an example of the application of the probabilistic interpretation (Section 4.2).

### 4.1 Applications

#### 4.1.1 NNs with unbalanced data

Datasets with unbalanced data are one of the most common problems in ML. However, it is possible to introduce constraints given by context-knowledge so as to mitigate the effect of unbalanced classes.

Sangalli et al. (2021) proposed to see the training of a DNN for binary classification under conditions of class imbalance as a constrained optimization problem. They consider the medical imaging context where applications with data imbalance are ubiquitous (Litjens et al., 2017) and some types of errors are more severe than others. For example, in a diagnosis application, discarding a cancer case as healthy (False Negative) is more costly than classifying a healthy subject as having cancer (False Positive). For this reason, networks working in the medical field generally tend to have high True Positive Rates (TPRs). The authors define a constraint, using the Mann-Whitney statistics (Mann and Whitney, 1947), to maximize the AUC, but to asymmetrically favor reducing False Positives in the presence of high TPR (or low False Negative Rates). They then use the Augmented Lagrangian Method (ALM) (Bertsekas, 1997).

The optimization problem they solve is defined as Equation (15):


(15) $$\begin{array}{l} \begin{array}{c} \arg\underset{\theta }{\min}F\left(\theta \right)\\ \text{subject to}:\\ \overset{\left|N\right|}{\underset{k=1}{\sum }}\max(0,-(f_{\theta }\left(x_{j}^{p}\right)-f_{\theta }\left(x_{k}^{n}\right))+\delta )=0,j\in \left\{1,...,|P|\right\} \end{array} \end{array}$$

where, *f*_θ_(*x*) indicates output probability of DNN on input *x*, and $P=\left\{x_{1}^{p},\ldots ,x_{\left|P\right|}^{p}\right\}$ is the set of positive samples and $N=\left\{x_{1}^{n},\ldots ,x_{\left|N\right|}^{n}\right\}$ is the set negative samples.

The constraint states that the output of the NN for each sample of the positive class should be larger than the outputs of all of the negative samples by a margin δ. Furthermore, satisfying the constraint would directly guarantee maximal AUC.

**Figure 2:**
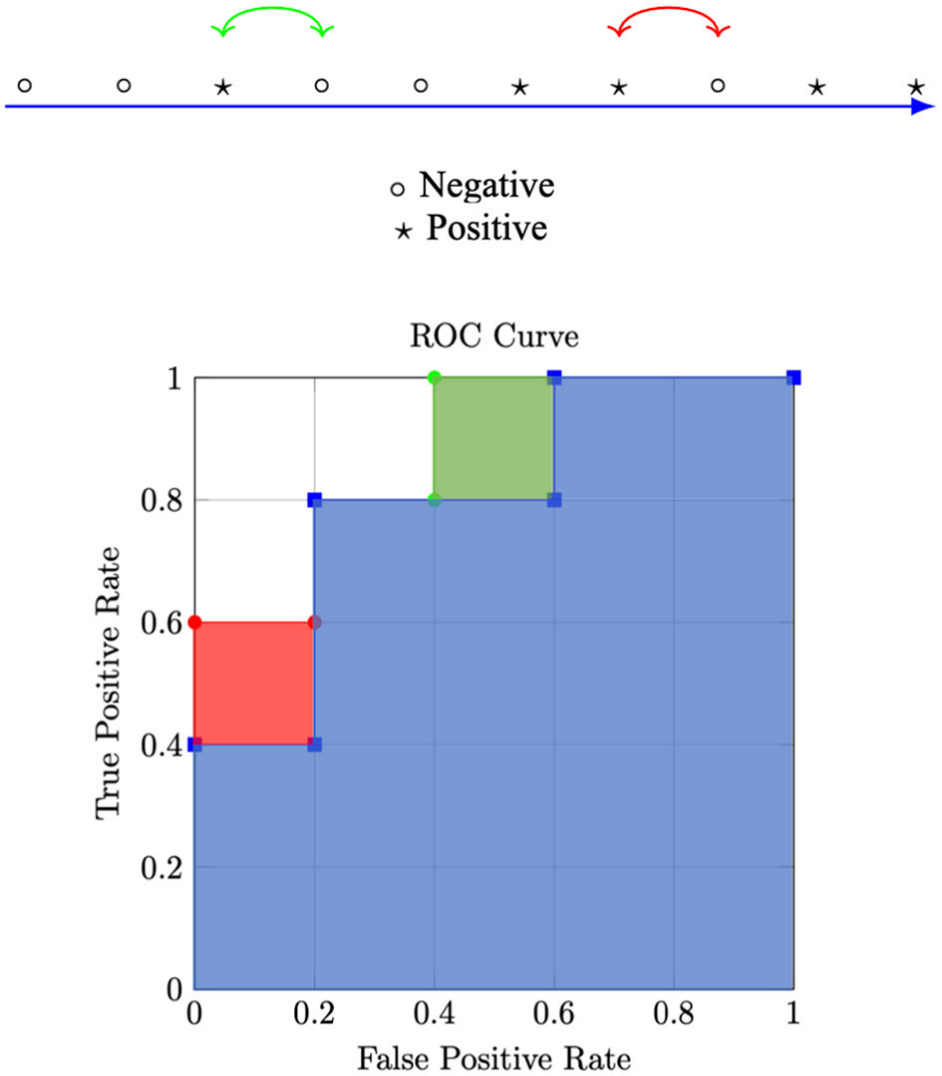
Toy example illustrating different optimizations. This Figure is a reelaboration of a Figure from Sangalli et al. (2021).

Figure 2 shows a toy example. The upper part shows 10 data samples sorted with respect to the output of the classifier, i.e., samples on the right are assumed to produce a higher output than those on the left. The blue area in the lower part of the figure shows the AUC for the toy data samples. Let us consider two different approaches to increase the AUC. Using standard optimization with binary cross entropy as the loss function, we would add the red box while solving the problem in Equation (15) via the method of Lagrange multipliers results in addition to the green box to the blue area. Both optimizations lead to the exacts same improvement in AUC, but only the addition of the green box reduces the FPR while maintaining the same TPR. Two possible extensions for multiclass classification are also proposed in Sangalli et al. (2021). The authors also perform an extensive evaluation of constraints for binary and multiclass image classification problems on both computer vision and medical imaging datasets by simulating different class imbalance ratios. They obtained results showing that constraints improve baseline performance in most cases of both binary and multiclass classification experiments.

#### 4.1.2 OOD detection with constrained networks

Katz-Samuels et al. (2022) proposed a framework for out-of-distribution (OOD) detection in the wild, dubbed WOODS (Wild OOD detection sans-Supervision). In this approach, a pre-trained model to perform in-distribution (ID) classification is deployed in the “wild” open world, where it will encounter large amounts of unlabeled ID and OOD data. In WOODS, the model can be tuned using the “wild” data to perform accurate OOD detection and ID classification.

The authors use wild data because it can be found in huge quantities, can be collected at low cost at the time of implementing an ML system, and often corresponds better to the actual distribution than data collected offline. However, it is difficult to exploit because it is naturally composed of examples of both ID and OOD data.

WOODS is based on constrained optimization and solves it through the Augmented Lagrangian method applied to deep neural networks.

Let *X* = ℝ^*d*^ denote the input space and *Y* = {1, ..., *K*} denote the label space. We assume access to the labeled training set $D_{in}^{train}={\left\{\left(x_{i},y_{i}\right)\right\}}_{i=1}^{n}$, drawn i.i.d. from the joint data distribution *P*_*XY*_. Let ℙ_in_ denote the marginal distribution on *X*, which is also referred to as the ID and ℙ_out_ denote different distribution on samples that the model has not been exposed to during training, which is also referred to as the OOD. Let $f_{\theta }:X\mapsto {\mathbb{R}}^{\left|Y\right|}$ denote a function for the classification task, which predicts the label of an input sample and *g*_ω_:*X*↦{in, out} as the function for OOD detection. WOODS uses the Huber contamination model (Huber, 1992) to model the marginal distribution of the wild data (Equation 16):


(16) $$\begin{array}{l} {\mathbb{P}}_{\text{wild}}:=\left(1-\pi \right){\mathbb{P}}_{\text{in}}+\pi {\mathbb{P}}_{\text{out}},\text{with}\pi \in \left(0,1\right] \end{array}$$

The objective can be described as follows (Equation 17):


(17) $$\begin{array}{l} \begin{array}{cc} \underset{\theta }{\mathrm{minimize}}\; & {𝔼}_{\text{x}\sim {\mathbb{P}}_{\text{wild}}}\left(𝟙\left\{g_{\theta }\left(\text{x}\right)=\text{in}\right\}\right)\\ s.t. & {𝔼}_{\text{x}\sim {\mathbb{P}}_{\text{in}}}\left(𝟙\left\{g_{\theta }\left(\text{x}\right)=\text{out}\right\}\right)\leq \alpha \\ {𝔼}_{\left(\text{x},y\right)\sim {\mathbb{P}}_{\mathrm{XY}}}\left(𝟙\left\{f_{\theta }\left(\text{x}\right)\neq y\right\}\right)\leq \tau . \end{array} \end{array}$$

where 𝟙{·} is the indicator function, and α, τ ∈ [0, 1].

In other words, the authors aim to minimize the number of samples that are classified as ID by applying two constraints:

1. The error of declaring an ID data from ℙ_in_ as OOD must be low;
2. The multiclass classification model must maintain the best attainable (or nearly attainable) accuracy of a base classifier designed without an OOD detection requirement.

### 4.2 Application of the probabilistic interpretation

When the output of a model is structured, knowledge of the structure type (one-hot encoder, ranking, path graph, etc.) can be useful information to integrate in the training of a neural network. In Xu et al. (2018), define a semantic loss to calculate how close the network output is to satisfying a given constraint, thus exploiting the concepts seen in Section 2.4. The idea is to penalize networks that do not satisfy constraints on the output.

The semantic loss *L*^*s*^(α, *p*) is a function involving the propositional logic sentence α and a set of variables **X** = {*X*_1_, …, *X*_*n*_}. It also incorporates a probability vector *p* associated with these variables, where each element *p*_*i*_ represents the predicted probability for the variable *X*_*i*_ and corresponds to a single output from the neural network. The semantic loss is defined by the following equation:


(18) $$\begin{array}{l} L^{s}\left(\alpha ,p\right)\propto -log\underset{x⊧\alpha }{\sum }\underset{i:x⊧X_{i}}{\prod }p_{i}\underset{i:x⊧\neg X_{i}}{\prod }\left(1-p_{j}\right) \end{array}$$

Semantic loss is simply another regularization term that can be directly added to an existing loss function. More specifically, given some weight *w*, the new loss becomes (Equation 19):


(19) $$\begin{array}{l} existing\;loss+w\cdot semantic\;loss \end{array}$$

For example, for given the exactly-one constraint seen in Section 2.4, the semantic loss is given by Equation (20):


(20) $$\begin{array}{l} L^{s}\left(exactly-one,p\right)\propto -log\overset{n}{\underset{i=1}{\sum }}p_{i}\overset{n}{\underset{j=1,j\neq i}{\prod }}\left(1-p_{i}\right) \end{array}$$

In general, for arbitrary constraints, computing semantic loss using Equation (18) is computationally expensive. Therefore, advanced automated reasoning, particularly knowledge compilation, is required (Anderson, 1983).

The authors show that semantic loss is not effective enough for simple supervised classification problems. However, it is useful, provided the output domain is a sufficiently complex space. A prime example of this is the path graph problem, where the goal is to predict the shortest path and the constraint is for the output to be a valid path. By testing the same network with and without semantic loss, Xu et al. (2018) show that the accuracy is significantly improved for consistent and constrained paths.

## 5 Conclusion and future work

The integration of domain knowledge, expressible in the form of constraints, into deep neural networks has gained research interest in recent years. In this study, we showed several AutoML and NAS frameworks that use multi-objective optimization and discussed how they compare with the state-of-the-art. As future work, we proposed utilizing knowledge to integrate Symbolic DNN-Tuner with multi-objective functions. Then, we presented some approaches that use domain knowledge in NNs. In particular, we showed how this integration can be done using Lagrange multipliers, both for domain constraints and detection cases such as OOD detection. We also discussed semantic loss, which calculates how close the network output is to satisfying a given constraint.

In summary, by combining developments of both approaches, AutoML and constrained neural networks, we obtain several promising applications of constraints in neural systems, resulting in more robust learning capabilities for neural-symbolic systems.

## Author contributions

AB: Investigation, Writing – original draft, Writing – review & editing. MF: Conceptualization, Supervision, Writing – review & editing. EL: Methodology, Supervision, Writing – review & editing. FR: Methodology, Supervision, Writing – review & editing.
